# Acknowledgement to Reviewers of *Diseases* in 2018

**DOI:** 10.3390/diseases7010006

**Published:** 2019-01-11

**Authors:** Diseases Editorial Office

**Affiliations:** MDPI, St. Alban-Anlage 66, 4052 Basel, Switzerland

Rigorous peer-review is the corner-stone of high-quality academic publishing. The editorial team greatly appreciates the reviewers who contributed their knowledge and expertise to the journal’s editorial process over the past 12 months. In 2018, a total of 107 papers were published in the journal, with a median time to first decision of 14 days and a median time to publication of 33 days. The editors would like to express their sincere gratitude to the following reviewers for their cooperation and dedication in 2018:

Aasvang, Gunn MaritLondono, BerlinAddington, CarolineLosurdo, GiuseppeAhlenstiel, ChantelleLozano, Jose ManuelAlexandre, MezentsevLu, ZhanpingAllen, HerbertLu, YufengAntoñanzas, FernandoLuca, MariaArndt, Patrick G.Luca, AdrianAthyros, VasiliosLund, Elizabeth K.Bachanova, VeronikaMaclellan, Reid A.Bakillah, AhmedMadeira De Carvalho, Luis ManuelBakonyi, TamásMaeder, Micha TobiasBalan, MurugabaskarMaher, PamelaBandea, ClaudiuMaiorano, EugenioBaragetti, AndreaMalfeito Ferreira, ManuelBasini, GiuseppinaMammadova-Bach, ElminaBates, John T.Mammola, Caterina LoredanaBattino, MaurizioMarengo, BarbaraBattistelli, CeciliaMarlicz, WojciechBeckman, JoanMartínez Álvarez, José AscenciónBertrand, GéraldMartini, MaurizioBodet, CharlesMasalova, Olga V.Borgstahl, Gloria E. O.Matouskova, PetraBortkiewicz, AlicjaMatsuo, MuneakiBoyanova, LyudmilaMatsuura, BunzoBrameld, JohnMcBride, Devin W.Brooke, JoanneMcCarty, CatherineBudzi, DorothMcCormack, Joseph G.Buigues, CristinaMcCown, WilliamCapasso, AnnaMei, Ya-FangCarneiro, Sueli Coelho Da SilvaMendez-Sanchez, NahunCastell Escuer, MargaridaMeschini, StefaniaCauli, OmarMesquita, JoãoCavé, ChristianMetwally, MayadaCécile, OuryMetz, Stefan W.Chalah, MoussaMilavetz, BarryChamcheu, Jean ChristopherMiranda, CristobalChang, Chien-ChungMitra, Amal K.Chaudhary, UjwalMlocicki, DanielChen, WenchunMonroy, FernandoChen, Yann-JangMoreno, MargaritaChendrasekhar, AkellaMou, HaiweiChien, Yin-HsiuMukaetova-Ladinska, Elizabeta BCicero, Arrigo Francesco GiuseppeMukhopadhyay, TuliCimmino, GiovanniMuto, AkiraClement, SophieNanno, MasanobuCorreia Santos, NadineNaruishi, KojiCoussens, ScottNavarro-Martínez, RutCraig-Kuhn, Megan ClareNemeth, LynneCruz-Sosa, F.Nifli, Artemissia-PhoebeCullen, PaulNissapatorn, VeeranootDanesh-Sani, Seyed AmirNodomi, SeishiroDawidowicz, A. L.Obeng-Gyasi, EmmanuelDe Berardis, DomenicoOlivier, Jean LucDe Geest, BartOmar, Syed HarisDe Pietro, GiuseppeOnyenwoke, Rob UDeevska, GerganaOon, Hazel H.Dehghannasiri, RoozbehOrtolano, SaidaDesselberger, UlrichPerez Gregorio, Maria RosaDevadas, KrishnakumarPérez-Prieto, DanielDi Fazio, PietroPerry, RachelDi Martino, GiuseppePeterson, JuliaDibaba, DanielPiche, Marie-EveDimopoulos, KonstantinosPinent, MontserratDing, YinyuanPistelli, FrancescoDomingo-Gonzalez, RacquelPlaza-Díaz, JulioDosoky, NouraPokorn, MarkoDou, XiaoguangPosada-Quintero, HugoDowner, BrianPovey, AndrewDu, Xiao-junProbst, YasmineDubińska-Magiera, MagdaProcházka, AlešEdwards, ErikaProkai, LaszloEilertsen, Karl-ErikRagusa, AndreaEspuny, Antoni CaminsRahman, Muhammad AzizEzhov, Marat V.Rajamani, SathishFernandez, Maria LuzRanzato, EliaFetissov, Sergueï O.Raposio, EdoardoFranconi, RosellaRather, Irfan AhmadFrisher, MartinRathore, Abhay PSFukuda, YoshiharuReid, St PatrickFuruhashi, KazukiRen, ZefangGaddam, Ravinder ReddyReyes-Sandoval, ArturoGarus-Pakowska, AnnaReynoso, ElenaGhareh Baghi, ArashRisbano, MichaelGoroll, Allan H.Rodenbeck, Stacey DineenGorska-Ponikowska, MagdalenaRossi, MiriamGray, AndrewRosso, NataliaGreen, James A.Rozsival, PavelGreenberger, Joel S.Rudolph, BryanGuido, Luis F.Saera-Vila, AlfonsoGyawali, PradipSalvati, BrunoHanai, Jun-ichiSantulli, GaetanoHarvey, AdamSaraux, AlainHassan, AmanySassi, YassineHead, MichaelSatake, HironagaHegde, Muralidhar L.Sato, Marcello OtakeHeggermont, WardSeeman, Mary V.Higa, Gerald M.Shepherd, SamanthaHingorani, DinaSherer, NathanHioki, HirofumiSherwin, LeeAnne B.Hodge, AllisonShi, JiaqiHodgson, IanShieh, Tzong-MingHoffman, RichardShim, Joon W.Huang, Guan-JhongShipman, KateHubková, BeátaSi, DongHung, Li-FangSiniscalco, DarioIchikawa, HiroshiSmith, Barry C.Italia, SalvatoreSong, ByeongwoonIvanov, Alexander VSong, ChunhuaJacova, ClaudiaSonntag, KaiJeckel, Kimberly MSoudeyns, HugoJerebtsova, MarinaSpassov, SashkoJohnston, SimonStahelin, RobertJordão, António ManuelStauber, Rudolf E.Just, NathalieStefani, LauraKabała-Dzik, AgataSubburaju, SivanKakisis, John D.Sun, XiaomingKam, Yiu WingTai, WanboKang, SinyoungTan, BKKang, Jae SeungTarantino, GiovanniKaranis, PanagiotisTeng, Ba-BieKarkhanis, VrajeshTeschke, RolfKarlin, BradleyToribio-Mateas, MiguelKeightley, CristinaTorquati, LucianaKim, Kyung-suTovoli, FrancescoKim, Hyung SikTrovato, GuglielmoKojima, MasamiTuriel, MaurizioKones, RichardUfnal, MarcinKonstandin, MathiasUnchwaniwala, NuruddinKonstantinidou, ValentiniUrbanelli, LorenaKosmas, Constantine E.Van Der Spoel, Aarnoud C.Koyama, ShinVan Hoek, BartKrajewska-Włodarczyk, MagdalenaVan Zanten, MalouKujawska, MałgorzataVarrica, DanielaKumar, BinodVautier, SimonKumar, MukeshVelez, ZéliaKurdowska, Anna K.Venkatesh, SiddarthKwan, Hiu-yeeVeraldi, StefanoLang, ChimVergara, DanieleLanghammer, BirgittaVinciguerra, ManlioLass, RichardVitetta, LuisLauschke, VolkerVogtmann, EmilyLava, Sebastiano A.G.Vozza, IoleLebaron, RichardWada, JunLedda, CaterinaWall-Medrano, AbrahamLedesma, Maria DoloresWalther, WolfgangLee, GihyunWang, ZenengLeibowitz, AvshalomWang, XiaoqinLeimanis, MaraWeinstein, PhilipLi, ZijianWeir, DawnLiang, YanWenner, YaroslavaLin, Gen-MinWoo, So-YounLin, Huey-JenXiang, YuLin, Chih-LiYasuda, KazukiLin, Liang-TzungYehuda, ShoenfeldLin, Ro-TingYi, TaoLiu, WeiYiin, Lih-MingLiu, Pei-YangYu, Chia-LiLiu, YuZhang, YuLombardi, RaffaellaZhu, Qiuyu MartinLonardo, Amedeo


